# Effects of supplementation of pomegranate processing by-products and waste cooking oils as alternative feed resources in broiler nutrition

**DOI:** 10.1038/s41598-022-25761-7

**Published:** 2022-12-08

**Authors:** Mohammad Ghasemi-Sadabadi, Yahya Ebrahimnezhad, Naser Maheri-Sis, Abdolahad Shaddel-Teli, Jamshid Ghiasi Ghalehkandi, Teun Veldkamp

**Affiliations:** 1 Department of Animal Science, Shabestar Branch, Islamic Azad University, Shabestar, East Azerbaijan Iran; 2 Wageningen University & Research, Wageningen Livestock Research, P.O. Box 338, 6700 AH Wageningen, The Netherlands

**Keywords:** Carbohydrates, Lipids, Biochemistry, Anatomy

## Abstract

Agricultural residues can be used as alternative feed sources in industrial chicken production. The impacts of different levels of pomegranate peel and waste cooking oil as an agricultural residue on broilers' nutrition were investigated. Results showed that the replacement of 8% pomegranate peel in diets decreased the growth performance of broilers. Supplementing 8% pomegranate peel in diets reduced apparent nutrient digestibility. The highest level of waste oil inclusion in broiler diets indicated negative impacts on apparent zmetabolizable energy and crude fat apparent nutrients digestibility. Broilers fed the diet containing 4% pomegranate peel had a higher *Lactobacillus* population. The results showed that the *Lactobacillus* population was lower in broilers fed 8% pomegranate peel powder and 4% waste oil in diets. The inclusion of 8% pomegranate peel powder in diets showed lower villus height and crypt depth in the duodenum, jejunum, and ileum. The inclusion of 4% pomegranate peel decreased the peroxide value (PV) of meat. Dietary inclusion of 4% waste oil raised the PV of meat. Alpha-tocopherol supplementation decreased the PV of meat. Finally, the results provide information that 4% of pomegranate peel and 4% waste oil could be used as an alternative feed ingredient in broiler diets without adverse effects.

## Introduction

It should be noted that feeding cost is one of the important factors in poultry industry. Therefore, nutritionists have attempted to improve productivity and decrease feedstuff’s cost. Hence, one way to reduce the prices of feed is to replace them with cheap items such as agricultural and food industry waste^1^. Accordingly, it appears that pomegranate peel is one of the agricultural residues that can be a suitable source of feedstuff for livestock^2^. Pomegranate has been cultivated widely from ancient times in tropical and subtropical areas. Pomegranate peel remains after pomegranate juice and pastes production. It should be noted that the peel of pomegranate constitutes up to 50% of the whole fruit^3^. Pomegranate peel contains a large number of high molecular weight phenolic compounds including tannins, proanthocyanidins, and complex polysaccharides such as ellagic acid, and gallic acid, which can have an antioxidant, antimicrobial, and anticancer effects in animals and human^4^. Researchers stated that the use of pomegranate peel in broiler diets improved the performance of broilers^3^. Also, Abbas et al.^5^ indicated that the addition of 4.5 and 7.5% pomegranate peel inclusion in diets improved the growth performance of quails.

The last studies revealed that pomegranate peel has nutritional value, which can be used as a cheap feed source in broiler diets^5^. The researchers indicated that the use of 4 and 7.5% pomegranate peel inclusion could be used as an alternative feed source and antioxidant in bird diets^2,5^.

Recently, phytochemicals or various active ingredients in plants have been isolated from different herb species and have been investigated for their effectiveness in animal health and promoting their products^6^. Bio-active ingredients of the plants are organic compounds obtained from the secondary metabolism of the plant divided into various groups: polyphenols (tannins), saponins, organ sulfur, and one of their approved significant attributes is their antimicrobial activity against yeasts, bacterium, and fungi^7^. It had noted that pomegranate peel had an antiviral potential. In this regard, the researchers demonstrated that pomegranate peel could prevent SARS-CoV-2 entry into host cells. Also, the researchers showed that pomegranate peel polyphenols had antiviral impacts against COVID-19^8^. The effectiveness of these compounds and especially polyphenols (tannins) and saponins were investigated for their antimicrobial activity and their effectiveness in gastrointestinal tract improvement^9^.

Waste vegetable oil (oxidized oil), obtained from food processing which is mostly produced due to lifestyle changes and increased consumption of fast food by human beings, may be used as a high-energy, and low-cost feed resource in broiler diets^2^. Some previous studies demonstrated that the waste oil could be supplemented in diets without any adverse effects for broilers. In contrast, some others observed that utilization of high peroxide value oil (oxidized oil) in diets has disadvantageous impacts on the health and performance of birds^10^. It seems that the supplementation of waste oil resulted in increased free radicals and oxidative stress in birds. It should be noted that malondialdehyde (MDA) is the second product of peroxidation that has a harmful influence on broiler health^11^. In general, waste oil generates free radicals which could have harmful consequences on the intestinal performance and health of broilers^12^. Reactive oxygen species (ROS) resulted in oxidative stress in the body, which harms broiler health and digestibility^11^. Pomegranate peel contains phenolic compounds such as tannins which can have antioxidant activity in broilers^13^. Also, it seems that the antioxidant characteristics of pomegranate peel reduced the adverse impacts of oxidative stress caused by oxidized oil^4^. The improvement in the global populations has caused a global revolution in food consumption models and enhanced requests for livestock products such as broiler chickens^14^. The higher demands in human life have led to a direct effect on increasing demand and production of livestock feedstuff which has inevitably placed increased pressure on limited resources^14^. Scientists indicated that pomegranate peel and waste vegetable oil as a by-product can replace in broiler diets. It seems that consuming this waste in poultry nutrition can diminish the disposal of this waste in the environment thus preventing environmental pollution. Pomegranate peel can also be a potential source of raw material for the animal feed industry. Generally, it has been hypothesized that utilizing these products can result in decreasing waste production and broiler industry diet cost. Also, it seems that pomegranate peel is one of the medicinal plants that had a strong antioxidant activity for poultry^2^. Hence, pomegranate peel and oxidized oil may play an important function as a new resource for broiler diets^15^. Nevertheless, a general investigation of the effects of these products on intestinal function and digestibility seems important. Therefore, the present study aimed to investigate the effects of using waste oils and pomegranate peel in diets on growth performance, intestinal function, apparent nutrient digestibility, and meat quality in broiler chickens.

## Materials and methods

### Animals, breeding, and nutrition

The research protocol has been approved by the Animal Care and Use Committee of the Islamic Azad University (93/987-2014) ^16^, The research protocol advises animal rights and welfare by ensuring minimal stress to animals. All procedures were carried out by the relevant research protocol. The experiment was carried out in keeping with the ARRIVE guidelines. The experiment was designed according to 3 × 3 × 2 factorial arrangement, with factors (i) the pomegranate peel powder (0, 4, and 8% in diets), (ii) the oxidized oil (0, 2, and 4% in diets) and (iii) the α-tocopherol (zero and 200 mg/kg). Hence, a total of 1080-day-old, male Ross 308 strain broiler chickens, were randomly allocated to 90-floor pens in a completely randomized design with 18 treatments and five replicates with twelve chicks in each replicate. The chicks were fed commercial diets during the first 10 days of the experiment. At the end of 10 days, the chicks were weighed and housed in 1.5 × 1.5 m floor pens each equipped with a pan feeder and manual drinker. Stocking density is based on the EU broiler welfare Directive (2007), 33 kg/m^2^. Birds received 22L:2D throughout the experiment period. Feed and water were available ad libitum. In this study, chicks received mash diets. All chicks were kept under management conditions according to the Ross 308 strain catalog. All chicks in the treatment groups were fed a grower (11–24 days) and finisher diet (25–49 days). All diets were formulated based on nutritional requirements proposed by the National Research Council^17^ (Tables 1 and 2). The composition (CP EE and CF) of all diets was measured by the AOAC method^18^ (Table 3). The basal diets in this experiment were corn and soybean meal and according to the treatments, different percentages of pomegranate peel powder, oxidized soybean oil, and α-tocopherol were added to the basal diets. The commercial α-tocopherol in this experiment was provided by Science Laboratories^®^ (Info@Sclabs.ir), Iran, and is a powder α-tocopherol supplement that consists of 5500 IU α-tocopherol.

**Table 1:** Ingredient composition and calculated chemical composition of the diets (11–24 days).

Ingredient (%)	Dietary treatment																	
	1	2	3	4	5	6	7	8	9	10	11	12	13	14	15	16	17	18
Corn grain	52.1	52.1	52.6	52.6	51.3	51.6	59.1	59.1	54	54.2	53.8	54	48.9	48.9	48.7	48.7	49.4	49.4
Soybean meal (44% CP)	31.8	31.8	33	33	29.7	29.7	32.7	32.7	30.2	30.2	25.8	25.8	31.9	31.9	32.6	32.6	33.7	33.7
Wheat	4.65	4.65	2.22	2.22	0.69	0.42	0.37	0.37	3.44	3.17	0.6	0.33	5	5	3	3	0	0
Pomegranate peel	0	0	4	4	8	8	0	0	4	4	8	8	0	0	4	4	8	8
Waste oil	0	0	0	0	0	0	2	2	2	2	2	2	4	4	4	4	4	4
α-tocopherol	0	0.02	0	0.02	0	0.02	0	0.02	0	0.02	0	0.02	0	0.02	0	0.02	0	0.02
Soybean oil	3	3	3	3	3	3	0	0	0	0	0	0	0	0	0	0	0	0
Gluten meal	0	0	0	0	2.28	2.3	0	0	1.42	1.44	4.53	4.55	0	0	0	0	0	0
Wheat bran	3	3	0	0	0	0	0	0	0	0	0	0	4	4	2.5	2.5	0	0
Dicalcium phosphate	1.58	1.58	1.58	1.58	1.6	1.6	1.59	1.59	1.6	1.6	1.63	1.63	1.58	1.58	1.57	1.57	1.57	1.57
Oyster sell	1.18	1.18	1.15	1.15	1.13	1.13	1.18	1.18	1.16	1.16	1.14	1.14	1.18	1.18	1.15	1.15	1.11	1.11
Bicarbonate Na	0.41	0.41	0.39	0.39	0.43	0.43	0.41	0.41	0.43	0.43	0.48	0.48	0.4	0.4	0.39	0.39	0.4	0.4
dl-methionine^a^	0.38	0.38	0.38	0.38	0.37	0.37	0.37	0.37	0.37	0.37	0.36	0.36	0.38	0.38	0.39	0.39	0.39	0.39
l-lysine HCl^b^	0.37	0.37	0.35	0.35	0.44	0.44	0.36	0.36	0.42	0.42	0.54	0.54	0.37	0.37	0.35	0.35	0.33	0.33
l-threonine^c^	0.18	0.18	0.18	0.18	0.2	0.2	0.17	0.17	0.19	0.19	0.22	0.22	0.18	0.18	0.18	0.18	0.19	0.19
Vitamin premix^d^	0.25	0.25	0.25	0.25	0.25	0.25	0.25	0.25	0.25	0.25	0.25	0.25	0.25	0.25	0.25	0.25	0.25	0.25
Mineral premix^e^	0.25	0.25	0.25	0.25	0.25	0.25	0.25	0.25	0.25	0.25	0.25	0.25	0.25	0.25	0.25	0.25	0.25	0.25
Common salt	0.08	0.08	0.09	0.09	0.06	0.06	0.08	0.08	0.06	0.06	0.02	0.02	0.08	0.08	0.09	0.09	0.1	0.1
K_2_SO_4_	0	0	0	0	0.16	0.16	0	0	0.1	0.1	0.31	0.31	0	0	0	0	0	0
Salinomycin	0.05	0.05	0.05	0.05	0.05	0.05	0.05	0.05	0.05	0.05	0.05	0.05	0.05	0.05	0.05	0.05	0.05	0.05
Inert	0.67	0.65	0.49	0.47	0.01	0	1.09	1.07	0	0	0	0	1.37	1.35	0.44	0.42	0.18	0.16
**Calculated chemical composition**																		
Apparent metabolizable energy (MJ/kg)	12.2	12.2	12.2	12.2	12.2	12.2	12.2	12.2	12.2	12.2	12.2	12.2	12.2	12.2	12.2	12.2	12.2	12.2
Calcium (%)	0.87	0.87	0.87	0.87	0.87	0.87	0.87	0.87	0.87	0.87	0.87	0.87	0.87	0.87	0.87	0.87	0.87	0.87
Available phosphorus (%)	0.43	0.43	0.43	0.43	0.43	0.43	0.43	0.43	0.43	0.43	0.43	0.43	0.43	0.43	0.43	0.43	0.43	0.43
Sodium (%)	0.16	0.16	0.16	0.16	0.16	0.16	0.16	0.16	0.16	0.16	0.16	0.16	0.16	0.16	0.16	0.16	0.16	0.16
Chlorine (%)	0.16	0.16	0.16	0.16	0.16	0.16	0.16	0.16	0.16	0.16	0.16	0.16	0.16	0.16	0.16	0.16	0.16	0.16
Potassium (%)	0.84	0.84	0.83	0.83	0.83	0.83	0.83	0.83	0.83	0.83	0.83	0.83	0.84	0.85	0.84	0.84	0.84	0.83
Linoleic acid (%)	2.76	2.76	2.72	2.72	2.68	2.69	2.39	2.39	2.26	2.26	2.24	2.25	3.19	3.19	3.16	3.16	3.14	3.14
Lysine (%)	1.3	1.3	1.3	1.3	1.3	1.3	1.3	1.3	1.3	1.3	1.3	1.3	1.3	1.3	1.3	1.3	1.3	1.3
Methionine + cysteine (%)	1	1	1	1	1	1	1	1	1	1	1	1	1	1	1	1	1	1
Threonine (%)	0.9	0.9	0.9	0.9	0.9	0.9	0.9	0.9	0.9	0.9	0.9	0.9	0.9	0.9	0.9	0.9	0.9	0.9
Tryptophan (%)	0.27	0.27	0.27	0.27	0.25	0.25	0.27	0.27	0.26	0.26	0.24	0.24	0.28	0.28	0.28	0.28	0.27	0.27

^a^dl-methionine, feed grade 99% (Evonik methionine), ^b^l-lysine feed grade 98.5% (Alborz Gostar Darou Co), ^c^l-threonine, feed grade 98.5% (Evonik methionine). ^d^Vitamin mixture provided per kilogram of diet: 15,000 IU vitamin A (retinol), 3750 IU vitamin D3 (cholecalciferol), 20.20 mg vitamin E (α-tocopherol), 3.7 mg vitamin K3 (menadione), 3 mg vitamin B1 (thiamine), 7.5 mg of vitamin B2 (riboflavin), 55 mg vitamin B3 (niacin), 4.5 mg of vitamin B6 (pyridoxine), 1 mg vitamin B7 (biotin), 15 mg vitamin B12 (cobalamin), and 11.5 mg of vitamin B5 (pantothenic acid). ^e^Mineral mixture provided per kilogram of diet: 50 mg Mn (manganese), 47 mg Zn (zinc oxide), 25 mg Fe (iron), 7.5 mg Cu (copper sulfate), 2.6 mg I (iodine), and 0.40 mg Se (selenium).

**Table 2:** Ingredient composition and calculated chemical composition of the diets (25–49 days).

Ingredient (%)	Dietary treatment																	
	1	2	3	4	5	6	7	8	9	10	11	12	13	14	15	16	17	18
Corn grain	62.6	62.6	60.5	60.5	57.9	57.9	65.4	65.4	63	63	60.2	60.2	59.9	59.9	57.7	57.7	55.6	55.6
Soybean meal (44% CP)	27.7	27.7	27.7	27.7	23.9	23.9	27.2	27.2	25	25	20	20	28.3	28.3	28.2	28.2	27.9	27.8
Pomegranate peel	0	0	4	4	8	8	0	0	4	4	8	8	0	0	4	4	8	8
Waste oil	0	0	0	0	0	0	2	2	2	2	2	2	4	4	4	4	4	4
α-tocopherol	0	0.02	0	0.02	0	0.02	0	0.02	0	0.02	0	0.02	0	0.02	0	0.02	0	0.02
Soybean oil	3	3	3	3	3	3	0	0	0	0	0	0	0	0	0	0	0	0
Gluten meal	0	0	0	0	2.48	2.48	0	0	1.44	1.44	4.76	4.74	0	0	0	0	0	0
Dicalcium phosphate	1.46	1.46	1.45	1.45	1.47	1.47	1.46	1.46	1.47	1.47	1.5	1.5	1.47	1.47	1.45	1.45	1.45	1.45
Oyster sell	1.01	1.01	0.99	0.99	0.97	0.97	1.02	1.02	1	1	0.98	0.98	1.01	1.01	0.98	0.98	0.96	0.96
Bicarbonate Na	0.32	0.32	0.31	0.31	0.35	0.35	0.33	0.33	0.35	0.35	0.41	0.4	0.32	0.32	0.31	0.31	0.34	0.35
dl-methionine^a^	0.33	0.33	0.33	0.33	0.32	0.32	0.32	0.32	0.32	0.32	0.3	0.3	0.33	0.33	0.33	0.33	0.35	0.35
l-lysine HCl^b^	0.32	0.32	0.32	0.32	0.42	0.42	0.33	0.33	0.39	0.39	0.53	0.53	0.31	0.31	0.31	0.31	0.4	0.42
l-threonine^c^	0.15	0.15	0.16	0.16	0.18	0.18	0.15	0.15	0.17	0.17	0.2	0.2	0.15	0.15	0.15	0.15	0.17	0.17
Vitamin premix^d^	0.25	0.25	0.25	0.25	0.25	0.25	0.25	0.25	0.25	0.25	0.25	0.25	0.25	0.25	0.25	0.25	0.25	0.25
Mineral premix^e^	0.25	0.25	0.25	0.25	0.25	0.25	0.25	0.25	0.25	0.25	0.25	0.25	0.25	0.25	0.25	0.25	0.25	0.25
Common salt	0.25	0.25	0.25	0.25	0.22	0.22	0.24	0.24	0.22	0.22	0.18	0.18	0.25	0.25	0.25	0.25	0.23	0.22
K_2_SO_4_	0	0	0	0	0.14	0.14	0	0	0.07	0.07	0.3	0.3	0	0	0	0	0	0
Salinomycin	0.05	0.05	0.05	0.05	0.05	0.05	0.05	0.05	0.05	0.05	0.05	0.05	0.05	0.05	0.05	0.05	0.05	0.05
Inert	2.18	2.16	0.43	0.41	0.03	0.01	0.98	0.96	0.02	0	0.02	0.03	3.36	3.34	1.65	1.63	0	0
**Calculated chemical composition**																		
Apparent metabolizable energy (MJ/kg)	12.5	12.5	12.5	12.5	12.5	12.5	12.5	12.5	12.5	12.5	12.5	12.5	12.5	12.5	12.5	12.5	12.5	12.5
Calcium (%)	0.8	0.8	0.8	0.8	0.8	0.8	0.8	0.8	0.8	0.8	0.8	0.8	0.8	0.8	0.8	0.8	0.8	0.8
Available phosphorus (%)	0.39	0.39	0.39	0.39	0.39	0.39	0.39	0.39	0.39	0.39	0.39	0.39	0.39	0.39	0.39	0.39	0.39	0.39
Sodium (%)	0.2	0.2	0.2	0.2	0.2	0.2	0.2	0.2	0.2	0.2	0.2	0.2	0.2	0.2	0.2	0.2	0.2	0.2
Chlorine (%)	0.25	0.25	0.25	0.25	0.25	0.25	0.25	0.25	0.25	0.25	0.25	0.25	0.25	0.25	0.25	0.25	0.25	0.25
Potassium (%)	0.74	0.74	0.74	0.74	0.74	0.74	0.74	0.74	0.73	0.73	0.73	0.73	0.74	0.74	0.74	0.74	0.73	0.73
Linoleic acid (%)	2.92	2.92	2.88	2.88	2.81	2.81	2.5	2.5	2.44	2.44	2.36	2.35	3.35	3.35	3.3	3.3	3.25	3.25
Lysine (%)	1.15	1.15	1.15	1.15	1.15	1.15	1.15	1.15	1.15	1.15	1.15	1.15	1.15	1.15	1.15	1.15	1.21	1.22
Methionine + cysteine (%)	0.9	0.9	0.9	0.9	0.9	0.9	0.9	0.9	0.9	0.9	0.9	0.9	0.9	0.9	0.9	0.9	0.91	0.91
Threonine (%)	0.8	0.8	0.8	0.8	0.8	0.8	0.8	0.8	0.8	0.8	0.8	0.8	0.8	0.8	0.8	0.8	0.8	0.8
Tryptophan (%)	0.24	0.24	0.24	0.24	0.22	0.22	0.24	0.24	0.22	0.22	0.2	0.2	0.24	0.24	0.24	0.24	0.23	0.23

^a^dl-methionine, feed grade 99% (Evonik methionine), ^b^L-lysine feed grade 98.5% (Alborz Gostar Darou Co), ^c^l-threonine, feed grade 98.5% (Evonik methionine). ^d^Vitamin mixture provided per kilogram of diet: 15,000 IU vitamin A (retinol), 3750 IU vitamin D3 (cholecalciferol), 20.20 mg vitamin E (α-tocopherol), 3.7 mg vitamin K3 (menadione), 3 mg vitamin B1 (thiamine), 7.5 mg of vitamin B2 (riboflavin), 55 mg vitamin B3 (niacin), 4.5 mg of vitamin B6 (pyridoxine), 1 mg vitamin B7 (biotin), 15 mg vitamin B12 (cobalamin), and 11.5 mg of vitamin B5 (pantothenic acid). ^e^Mineral mixture provided per kilogram of diet: 50 mg Mn (manganese), 47 mg Zn (zinc oxide), 25 mg Fe (iron), 7.5 mg Cu (copper sulfate), 2.6 mg I (iodine), and 0.40 mg Se (selenium).

**Table 3:** Composition of the diets analyzed nutrient contents given to broilers chickens.

Analyzed composition of diets	Dietary treatment																	
	1	2	3	4	5	6	7	8	9	10	11	12	13	14	15	16	17	18
**11–24 d**																		
Crude protein (%)	20.2	20.1	20.2	20.2	20.2	20.1	20.2	20.2	20.2	20.1	20.2	20.2	20.2	20.1	20.2	20.2	20.2	20.1
Ether extract (%)	2.4	2.45	2.38	2.36	2.34	2.4	2.53	2.48	2.47	2.5	2.52	2.49	2.36	2.39	2.29	2.35	2.28	2.25
Crude fiber (%)	3.79	3.88	4.35	4.26	4.71	4.65	3.56	3.61	4.15	4.11	4.55	4.61	3.82	3.93	4.45	4.61	4.22	4.89
**25–49 d**																		
Crude protein (%)	18.2	18.2	18.1	18.2	18.1	18.2	18.2	18.2	18.2	18.1	18.1	18.2	18.2	18.2	18.2	18.1	18.2	18.2
Ether extract (%)	2.59	2.62	2.61	2.52	2.56	2.59	2.65	2.69	2.68	2.58	2.63	2.7	2.49	2.45	2.37	2.47	2.45	2.41
Crude fiber (%)	3.32	3.36	3.98	3.92	4.45	4.41	3.31	3.37	3.91	3.87	4.25	4.19	3.29	3.34	4	3.95	4.58	4.63

### Pomegranate peel composition

Pomegranate peel was obtained from a pomegranate processes factory. Peel was dried under ambient conditions and then milled and prepared for the experiment. In addition, the proximate chemical composition of pomegranate peel (i.e. dry matter (DM), crude protein (CP), Ether extract (EE), crude fiber (CF), nitrogen-free extract (NFE), and ash) was measured according to AOAC^18^ methods. Furthermore, hydrolysable tannins and condensed tannins in pomegranate peel were obtained^15^. The essential amino acids in pomegranate peel such as methionine, lysine, and threonine were analyzed according to the method defined by the AOAC^18^ (2005) and Cooperative^19^ using HPLC and the modification of the PICO-TAG process. The chemical composition of pomegranate peel is shown in Table 2. The gross energy of the pomegranate peel was measured by an adiabatic bomb and is shown in Table 1^20^. The Sib bald method was used to determine the metabolizable energy of pomegranate peel^21^ (Table 4).

**Table 4:** Nutritive and chemical composition of pomegranate peel.

Item	Pomegranate peel
Gross energy (kcal/kg)	4478
Apparent metabolizable energy (AME, kcal/kg)	1835
Nitrogen-corrected apparent metabolizable energy (AME_n_, kcal/kg)	1833
True metabolizable energy (TME, kcal/kg)	2475
Nitrogen-corrected true metabolizable energy (TME_n_, kcal/kg)	2470
Dry matter (%)	91.6
Crude protein (%)	5.20
Crude fat (%)	1.42
Crude fiber (%)	17.9
Ash (%)	3.37
Nitrogen free extract (NFE) (%)	72.0
Lysine (%)	0.20
Methionine (%)	0.09
Threonine (%)	0.05
Arginine (%)	0.25
Hydrolysable tannins (mg tannic acid/g dry matter)	203
Condensed tannins (mg cyaniding acid/g dry matter)	6.05

### Oxidized oil preparation

First, conventional soybean oil was poured into a metal gallon container and boiled for 18 h at 180 °C with aeration (aquarium air pump). Oxidized soybean oil was stored at −20 °C without any additives to avoid additional oxidation. The fatty acid profile of oxidized and fresh soybean oil was examined by using the method described by the American Oil Chemists Society^22^. Also, the peroxide value (PV) and MDA of samples were analyzed^23^. The chemical compositions of experimental soybean oils (oxidized and fresh oil) are shown in (Table 5).

**Table 5:** Analysis of the chemical composition of experimental soybean oils.

Item	Normal soybean oil	Oxidized soybean oil (waste oil)
PV (meq/kg)	2.07	49.0
MDA (μg/kg)	3.53	56.1
AnV	2.09	198
**Fatty acid profile**		
Myristic acid, C14:0	0.18	0.63
Palmitic acid, C16:0	13.9	35.9
Palmitoleic acid, C16:1	0.13	0.26
Stearic acid, C18:0	4.10	20.8
Oleic acid, C18:1	24.2	25.1
Linoleic acid, C18:2	50.1	15.6
Linolenic acid, C18:3	6.20	1.00
Total PUFA	56.3	16.6
Total MUFA	24.3	25.3
Total SFA	18.1	56.6

Results are the average of four analyses ± SD.

*PV* peroxide value, *MDA* malondialdehyde, *AnV* anisidine value soybean oil was heated up for 18 h at 180 °C.

### Growth performance

The performance of broilers in terms of body weight gain (BWG), feed intake (FI), and feed conversion ratio (FCR) during the experimental period (11–49 days) were determined.

### Apparent nutrient digestibility

Broilers received the experimental diets with the inclusion of 0.3% of indicator chromium oxide (Cr_2_O_3_) at 46 days old for three days. At the end of the experiment, two broilers from each pen were slaughtered via cervical dislocation, and ileal contents were collected for nutrient digestibility measurement. Each sample (ileal contents) was homogenized, pre-dried at 55 °C, ground to 1 mm screen, and examined. Concentrations of chromium oxide in the samples, as well as the indigestibility factor, were calculated according to formulas defined by Sakomura and Rostagno^24^. The digestibility of DM, crude protein, crude fat, gross energy, and chromium oxide concentration, was determined by the method as described. Also, the AME digestibility was measured by the methods of Scott and Boldaji^25^.

### Gut microbial population

At the end of the experiment, two broilers from each pen were randomly selected for the determination of intestinal microbial population. For intestinal microflora estimation, the primary one gram of the sample of the intestinal content from each broiler was diluted with 9 ml of 0.9% saline solution and combined on a vortex. In the following phase, 0.1 ml of proper dilutions of ileum were cultured in Violet Red Bile Agar (VRBA) for investigating coliform bacteria. Also, Rogossa Agar (MRSA) was utilized for *lactobacilli* bacteria. The *lactobacilli* and coliform bacteria cultures were conducted in dilutions of 10^–5^ and 10^–4^, respectively^26^. Besides, the anaerobic bacteria (total anaerobic bacteria) were counted by Reinforced Clostridial agar. It should be stated that all the phases continued near the flame until environmental pollution was eliminated. Bacterial colony forming units (CFU) were counted utilizing a colony counter. Finally, the bacteria counts were displayed as log_10_ colony-forming units per g of digestal (log_10_ CFU g^−1^)^16^.

### Gut morphology

On the last day of the experiment, and before slaughtering the birds, all of them starved for five hours and the feeding troughs were removed from the pens. Two broilers from each pen was slaughtered and subsequently, 5 cm length of intestine was sampled from the duodenum, jejunum, and ileum of it. The intestine samples were washed with sodium phosphate buffer and fixed by Clark stabilizer solution. All of the intestine samples were prepared for microscopic investigation later staining with Periodic Acid Schiff Solution by separating the muscular layer and making the lamella. Ultimately, the villi height and the crypt depth were estimated according to the process of Ghasemi-Sadabadi et al.^6^.

### Meat quality

At the end of the experiment, two broilers from each pen were slaughtered and the meat was trimmed and prepared for fatty acid profile and peroxide value measurement. The breast muscle was trimmed and stored at − 20 °C. Next, the meat sample (50 g) was mixed in a blender with 150 ml of Chloroform-methanol (Merck) in a 1:2 ratio for a minute. Then, 50 mL of sodium chloride (0.88%) was added to the mixture. Potassium chloride was used for dewatering, the aqueous phase was collected and methanol- potassium chloride 0.88% (v/v: 1/1) was mixed with the aqueous phase. The final solution was dried at 35 °C, after that, the residual solvent was evaporated under nitrogen gas pressure to obtain pure oil. Eventually, the fat was obtained for the measurement of peroxidation. To measure meat peroxidation, chloroform–acetic acid (25 ml) was added to one g of extracted fat in a ratio of 2:3. Then, one mL of potassium iodide was added to the mixture, which was stored for five min in the dark. The mixture was tittered with normal 1% potassium thiosulfate^6^. Peroxidation value was calculated based on the amount of peroxide (meq/kg extracted fat), according to the American Oil Chemists Society^22^. The fatty acid profile of the meat was analyzed by gas chromatography of the method as described by Ghasemi-Sadabadi^6,27^.

### Statistical analysis

Statistical analyses were performed using SAS (2003) software. The data were analyzed using a 3 × 3 × 2 factorial arrangement design. Significant differences between treatments were separated using the Tukey range test at (p < 0.05).

Data following model:

$${\text{Y}}_{{{\text{ijk}}}} = \, \mu \, + {\text{ P}}_{{\text{i}}} + {\text{ O}}_{{\text{j}}} + {\text{ V}}_{{\text{k}}} + {\text{ P}}_{{\text{i}}} {\text{O}}_{{\text{j}}} + {\text{ P}}_{{\text{i}}} {\text{V}}_{{\text{k}}} + {\text{ O}}_{{\text{j}}} {\text{V}}_{{\text{k}}} + {\text{ P}}_{{\text{i}}} {\text{O}}_{{\text{j}}} {\text{V}}_{{\text{k}}} + {\text{ E}}_{{{\text{ijk}}}}$$

Y_ijk_ is the dependent variable; μ is the overall mean; P_i_ is the effect of different pomegranate peel; O_j_ is the effect of waste oil; V_k_ of is the effect of α-tocopherol; P_i_O_j_ is the interaction of pomegranate peel and waste oil; P_i_V_k_ is the interaction of pomegranate peel and α-tocopherol; O_j_V_k_ is the interaction of waste oil and α- tocopherol; P_i_O_j_V_k_ is the interaction of pomegranate peel, waste oil, and α-tocopherol; E_ijk_ is the random error.

## Results and discussions

### Performance

The effects of using different levels of pomegranate peel, waste soybean cooking oil, and α-tocopherol in the diet on BWG, FI, and FCR in broiler chickens are shown in Table 6. The results of this research revealed that the use of pomegranate peel in diets had significant influences on BWG, FI, and FCR (p < 0.05). The data showed that the dietary addition of 8% pomegranate peel in broiler diets decreased BWG and FI compared to other groups (p < 0.05). No significant differences were observed in growth performance between the 4% pomegranate peel and the control group. In this experiment, growth performance during 11–49 days was not significantly affected by waste oil and α-tocopherol (p < 0.05). No significant interaction between pomegranate peel, waste oil, and α-tocopherol on growth performance results has been observed in this experiment. By examining the results obtained, it can be concluded that when incorporating 8% pomegranate peel into diets, the growth performance of broilers decreased in the experimental period. In similar experiments, researchers confirmed that the inclusion of pomegranate peel in diets diminished the performance of birds^4,15^. Similarly, the researchers stated that the addition of three g/kg pomegranate peel in broilers' diets decreased the growth performance^4,15^. One of the results of our research was that the inclusion of 4% pomegranate peel in diets had not significantly affected the growth performance of broilers. Also, Abbas et al.^5^ indicated that the addition of 4.5 and 7.5% pomegranate peel inclusion in diets did not affect the growth performance of Japanese quail. Generally, it seems that high amounts of tannins and CF of pomegranate peel impaired the growth performance of the birds^4^. Furthermore, Ghasemi-Sadabadi et al.^2^ stated that the pomegranate peel compounds such as CF and tannins may reduce gastrointestinal motility and consequently decrease the growth performance of birds. It has already been determined that the high amount of tannic acid reduces BWG and FI in poultry^28^. Past studies have shown that tannins can affect the physiological and biochemical systems of the body. In addition, tannins in the body lead to adverse nitrogen balances, growth inhibition, diminished intestinal absorption of sugars and amino acids, reduced immune response, and improved protein catabolism^29^. In this study, α-tocopherol did not affect growth performance. Contrary to this result, researchers indicated that vitamin E may be used in diets containing up to 6% of Distilled fatty acids for male quails because of the resulting betterment in nutrient utilization, growth performance, and meat quality^30^.

**Table 6:** The effects of using pomegranate peel, waste oil and α-tocopherol in diets on growth performance in broiler chickens (11–49 days).

Treatment			Traits		
			Body weight gain (g)	Feed intake (g)	Feed conversion ratio (g/g)
***Main effect***					
**Pomegranate peel (%)**					
0			2798^a^	5245^a^	1.87^b^
4			2780^a^	5210^a^	1.87^b^
8			2353^b^	4882^b^	2.08^a^
SEM			29.9	33.4	0.02
**Waste oil (%)**					
0			2668	5159	1.94
2			2658	5119	1.93
4			2606	5060	1.95
SEM			29.9	33.4	0.02
**α-tocopherol (mg/kg)**					
0			2624	5086	1.95
200			2664	5139	1.94
SEM			24.4	27.9	0.01
***Interaction effects***					
**Pomegranate peel × waste oil × α-tocopherol**					
0	0	0	2809	5276	1.88
0	0	200	2872	5373	1.87
0	2	0	2819	5231	1.86
0	2	200	2855	5307	1.86
0	4	0	2694	5106	1.89
0	4	200	2738	5179	1.89
4	0	0	2774	5201	1.87
4	0	200	2786	5246	1.88
4	2	0	2742	5203	1.90
4	2	200	2818	5225	1.85
4	4	0	2776	5195	1.87
4	4	200	2786	5190	1.87
8	0	0	2327	4881	2.10
8	0	200	2440	4974	2.04
8	2	0	2369	4864	2.06
8	2	200	2347	4886	2.08
8	4	0	2304	4816	2.09
8	4	200	2335	4868	2.09
SEM			73.5	81.9	0.06
**p-value**					
Pomegranate peel			0.0001	0.0001	0.0001
Waste oil			0.2922	0.1119	0.8776
α-tocopherol			0.2478	0.1745	0.6520
Pomegranate peel × waste oil			0.7388	0.7436	0.9910
Pomegranate peel × α-tocopherol			0.8999	0.8997	0.9669
Waste oil × α-tocopherol			0.9849	0.8112	0.9686
Pomegranate peel × waste oil × α-tocopherol			0.9161	0.9990	0.9353

*SEM* standard error of mean, *P-value* probability values.

^a^^:b^Means in columns with the same superscript do not differ significantly P < 0.05.

### Apparent nutrient digestibility

The results (Table 7) indicated that dietary inclusion of 8% pomegranate peel in broiler diets decreased the digestibility of DM, CP, CF, and apparent metabolizable energy (AME) compared to other groups (p < 0.05). In this study, the results demonstrated that broilers fed diets with 4% waste oil had significantly lower digestibility of CF and AME when compared to the control group (p < 0.05). There was no significant difference in nutrient digestibility between the control group and 2% waste oil treatments. According to the results, no significant interaction between treatments on results was observed in this experiment. In this study, DM, CP, CF, and AME digestibility of broilers decreased when incorporating 8% pomegranate peel into diets. The results were quite similar to the results of Saleh et al.^15^; Ghasem-Sadabadi et al.^2^. Also, our results agree with Akuru et al.^31^, who stated that the addition of pomegranate peel in diets reduced DM, CP, and CF digestibility in broilers. Similar effects were discussed by Saleh et al.^32^ for the nutrient digestibility of broilers fed high dietary levels of pomegranate peel. Consequently, it appears that the lower digestibility in the 8% pomegranate peel group in this study could be related to higher tannins and fiber contents^32^. Furthermore, previous examinations confirmed that the pomegranate peel contains a high amount of CF and tannin, which adversely affected absorption and digestion^33^. The high amount of tannins had destructive influences on the physiological and biochemical systems, which resulted in adverse nitrogen balances, lower intestinal absorption of sugars and amino acids, diminished immune response, and increased protein catabolism^29^. It has been mentioned that phenolic compounds such as tannins could be bonded to dietary and endogenous proteins such as digestive enzymes and proteins located in the gastrointestinal tract that reduced the apparent nutrient digestibility (Brenes et al.^34^). The reaction between the hydroxyl groups of polyphenol and the carbonyl groups of protein can explain the lower digestibility of protein in this study^35^. Similarly, researchers concluded that the use of high-tannin sorghum grain in broiler diets reduced protein digestibility^34^. The earlier reports had shown that the use of high polyphenol compounds in diets decreased fat absorption in rats and birds. Generally, tannins could bind to bile salts and cholesterol and reduce the digestibility of fat. Furthermore, trypsin, amylase, and lipase are the main digestive enzymes in birds that can be bonded by tannins and reduced digestibility of nutrients^32^. The results reveal a clear significant correlation between the waste oil and digestibility. According to the results, the supplementation of 4% waste oil decreased fat and AME digestibility. Our findings are in agreement with those of Kamran et al.^36^, who indicated that the addition of 4% waste oil in diets reduced digestibility. Tavárez et al.^10^ stated that the supplementation of waste oil in the diet diminished the growth performance of broilers. Moreover, feeding of waste oil results in lower digestibility of fat and energy in birds which is determined by the decreased capability of digestion^37^. It seems that parallel reduction of apparent CF digestibility, a reduced AME has been observed, indicating extensive fatty acid polymerization and a negative impact on the gastrointestinal tract^38^.

**Table 7:** The effects of using pomegranate peel, waste oil and α-tocopherol in diets on apparent nutrient digestibility and AME in broiler chickens.

Treatment			Nutrients			
			Dry matter (%)	Crude protein (%)	Crude fat (%)	AME (kcal/kg)
***Main effect***						
**Pomegranate peel (%)**						
0			70.9^ab^	70.7^ab^	69.8^a^	2770^a^
4			71.4^a^	71.3^a^	68.3^a^	2707^a^
8			65.7^b^	63.5^b^	60.1^b^	2344^b^
SEM			1.26	1.07	1.13	72.4
**Waste oil (%)**						
0			69.9	69.3	68.1^a^	2741^a^
2			70.3	69.1	67.1^ab^	2668^ab^
4			67.8	67.1	62.9^b^	2411^b^
SEM			1.26	1.07	1.13	72.4
**α-tocopherol (mg/kg)**						
0			68.3	68.0	65.8	2657
200			70.3	69.0	66.3	2557
SEM			1.03	0.87	0.92	59.1
***Interaction effects***						
**Pomegranate peel × waste oil × α-tocopherol**						
0	0	0	74.4	70.4	70.6	2911
0	0	200	73.3	70.7	73.0	2858
0	2	0	71.9	72.2	71.2	3110
0	2	200	73.0	71.4	71.3	2706
0	4	0	65.9	69.5	65.1	2745
0	4	200	67.0	69.9	67.3	2289
4	0	0	68.9	70.0	69.7	2898
4	0	200	72.0	73.7	70.3	2733
4	2	0	69.9	71.0	70.7	2635
4	2	200	72.0	72.1	68.1	2745
4	4	0	71.8	69.5	64.1	2662
4	4	200	73.5	71.5	66.8	2567
8	0	0	60.7	65.2	62.2	2544
8	0	200	70.0	64.5	62.9	2503
8	2	0	67.2	64.7	60.1	2296
8	2	200	67.7	64.5	61.3	2516
8	4	0	64.0	59.3	58.1	2109
8	4	200	64.4	63.0	56.0	2095
SEM			3.10	2.63	2.78	177
***P-*value**						
Pomegranate peel			0.0345	0.0005	0.0001	0.0054
Waste oil			0.5102	0.4743	0.0385	0.0428
α-tocopherol			0.2951	0.5146	0.7468	0.3610
Pomegranate peel × waste oil			0.4870	0.9697	0.9972	0.8764
Pomegranate peel × α-tocopherol			0.8082	0.8785	0.9128	0.8230
Waste oil × α-tocopherol			0.8059	0.8487	0.9190	0.3852
Pomegranate peel × waste oil × α-tocopherol			0.8578	0.9806	0.9462	0.8581

*SEM* standard error of mean, *P-value* probability values.

^a^^:b^ Means in columns with the same superscript do not differ significantly P < 0.05.

### Intestine microbial population

The effect of dietary pomegranate peel powder was significant (p < 0.05) on the *lactobacillus* and *coliform* bacteria populations from 11 to 49 days (Table 8). The highest *lactobacillus* count was observed in 4% pomegranate peel powder groups (p < 0.05). Although, broilers fed on diets including 4% of pomegranate peel powder had significantly lower coliform populations than other groups (p < 0.05). Although coliforms and the total bacteria population were not affected by dietary waste oil, significant differences were determined in the *lactobacilli* population of waste oil by linear contrast (p < 0.05) (Table 7). Hence, supplementation of 4% waste oil in the diet significantly decreased the *lactobacilli* population in broilers (p < 0.05). The *lactobacilli* bacteria populations were influenced by α-tocopherol supplementation within diets (p < 0.05) (Table 9). The highest *lactobacilli* bacteria population was found in the group containing 200 mg/kg α-tocopherol (p < 0.05). Besides, the lowest *lactobacilli* bacteria population was observed in the non-supplemented group. Further, an interaction was not found between dietary treatments on the intestinal microbial population in this experiment. The present study concluded the optimal level of pomegranate peel in diets required to achieve the best intestinal health in broilers at the end of the experiment. The use of 4% pomegranate peel powder in diets increased *lactobacilli* bacteria count in broilers, whereas broilers fed with 4% pomegranate peel powder had lower *coliform* bacteria count. In similar studies, researchers have reported that pomegranate peel increased beneficial microorganisms of the intestine such as *lactobacilli* bacteria by restraining the pathogens bacteria, which improved digestion and absorption of broilers^39^. Overall, researchers suggested that phenolic compounds have antimicrobial characteristics. It was mentioned that phenolic compounds through the adhesion of pathogenic microorganisms by the ‘lectin–receptor’ mechanism and inhibition of the incorporation of pathogenic bacteria to the mucosal layer decreased gut pathogenic bacteria counts^40^. The phenolic compounds played an antimicrobial role against pathogenic microorganisms such as *E.coli* bacteria^41^. In general, it has been noted that higher *lactobacillus* counts in the intestine were well correlated with lower *coliforms* and *salmonella* bacteria count, which improved broiler performance and health^16^. The development in the lactobacilli bacteria count in this study seems to be related to the reduction in pathogenic bacteria in the gut^16^. Similarly, the tannins reduce pathogenic bacteria; hinder harmful gut microbe's metabolism and the activities of harmful microbial enzymes by preventing oxidative phosphorylation^42^. The results showed that the use of 4% waste oil in diets decreased the *lactobacilli* bacteria counts in broilers. Also, the inclusion of the different levels of waste oil in diets did not affect coliform bacteria and the total bacteria population. In the same case, it has been demonstrated that oxidative stress harmed intestinal microflora, which increased pathogenic bacteria in the intestine^43^. Oxidative stress influences the gut epithelial cells and stimulates intestinal bacteria and lipopolysaccharide (LPS). It seems that LPS is understood to cause apoptosis and damage in different cells^44^. Also, the researchers stated that oxidative stress could have a destructive effect on the intestinal immune barrier that increased pathogenic bacteria and inflammatory infiltrate^12^. In the present study, the supplementation of 200 mg/kg α-tocopherol was shown to increase the *lactobacilli* population in broilers. In contrast, Dalia et al.^45^ reported that dietary 100 mg/kg α-tocopherol in broilers could not affect the caecal *lactobacilli* bacteria population. Results on the influence of α-tocopherol on microbial population are limited. Nevertheless, the researcher indicated that the decline in *Salmonella spp*. population due to α-tocopherol use unclear^45^. Hernken et al.^46^ concluded that α-tocopherol could affect the antioxidant and immunity response of broilers. The use of vitamin E has been shown to decrease some of the adverse effects of oxidative stress^47^. Therefore, it is well known that the antioxidants substances improved intestinal health and functioning due to a reduction in oxidation stress in the intestine^45^.

**Table 8:** The effects of using pomegranate peel powder, waste vegetable oil and α-tocopherol in diets on gut microbial population in broiler chickens.

Treatment			Parameters		
			*Lactobacillus* (log_10_ CFU g^−1^)	Coliform bacteria (log_10_ CFU g^−1^)	Total bacteria (log_10_ CFU g^−1^)
***Main effect***					
**Pomegranate peel (%)**					
0			4.78^ab^	5.54^ab^	6.86
4			5.34^a^	4.78^b^	6.74
8			4.62^b^	5.60^a^	6.89
SEM			0.3	0.22	0.23
**Waste oil (%)**					
0			5.22^a^	5.11	6.83
2			5.10^ab^	5.17	6.73
4			4.43^b^	5.64	6.94
SEM			0.30	0.22	0.23
**α-tocopherol (mg/kg)**					
0			4.59^b^	5.32	6.90
200			5.24^a^	5.29	6.76
SEM			0.25	0.19	0.19
***Interaction effects***					
**Pomegranate peel × waste oil × α-tocopherol**					
0	0	0	4.98	4.58	7.11
0	0	200	5.59	5.07	6.71
0	2	0	4.75	4.97	6.41
0	2	200	5.81	5.66	6.84
0	4	0	3.68	6.78	7.21
0	4	200	3.93	6.19	6.93
4	0	0	5.16	5.12	7.03
4	0	200	5.63	5.11	6.58
4	2	0	4.45	4.88	6.89
4	2	200	6.42	3.73	6.66
4	4	0	5.30	4.91	6.90
4	4	200	5.11	4.96	6.43
8	0	0	4.55	5.43	6.93
8	0	200	5.46	5.35	6.64
8	2	0	4.59	6.27	6.61
8	2	200	4.59	5.52	7.01
8	4	0	3.88	5.01	7.08
8	4	200	4.69	6.05	7.09
SEM			0.75	0.57	0.58
***P*-value**					
Pomegranate peel			0.0488	0.0295	0.8985
Waste oil			0.0213	0.2197	0.8325
α-tocopherol			0.0095	0.8977	0.6004
Pomegranate peel × waste oil			0.3176	0.0874	0.9610
Pomegranate peel × α-tocopherol			0.9572	0.6796	0.8125
waste oil × α-tocopherol			0.4928	0.6347	0.6674
Pomegranate peel × waste oil × α-tocopherol			0.3368	0.3814	0.9960

^a,b^^,c^Means in columns with the same superscript do not differ significantly P < 0.05.

*SEM* standard error of means, *P-value* probability values.

**Table 9:** The effects of using pomegranate peel powder, waste vegetable oil and α-tocopherol in diets on gut morphology (30, 60 and 90% of gut) in broiler chickens.

Treatment			Parameters								
			Villus height (μm)			Crypt depth (μm)			Villus/crypt		
			Duodenum	Jejunum	Ileum	Duodenum	Jejunum	Ileum	Duodenum	Jejunum	Ileum
***Main effect***											
**Pomegranate peel (%)**											
0			1712^a^	1222.^a^	847^a^	250	215^ab^	181	7.07^a^	5.68^a^	4.71^a^
4			1783^a^	1277^a^	834^a^	257	223^a^	184	7.01^ab^	5.76^a^	4.55^a^
8			1530^b^	1034^b^	659^b^	242	204^b^	172	6.29^b^	5.08^b^	3.85^b^
SEM			45.8	31.1	26.7	5.2	5.1	4.7	0.22	0.15	0.14
**Waste oil (%)**											
0			1744^a^	1272^a^	813	254	219	182	6.95^ab^	5.81^a^	4.46
2			1733^a^	1211^a^	796	247	220	183	7.11^a^	5.61^ab^	4.40
4			1548^b^	1040^b^	731	247	204	172	6.30^b^	5.10^b^	4.25
SEM			45.8	31.13	26.7	5.29	5.13	4.71	0.22	0.15	0.14
**α-tocopherol (mg/kg)**											
0			1681	1162	782	252	215	178	6.75	5.41	4.39
200			1669	1194	778	247	214	180	6.83	5.60	4.35
SEM			37.43	25.41	21.84	4.39	4.19	3.85	0.18	0.12	0.11
***Interaction effects***											
**Pomegranate peel × waste oil × α-tocopherol**											
0	0	0	1862	1347	940	263	226	183	7.24	5.96	5.09
0	0	200	1761	1360	881	249	223	187	7.88	6.12	4.71
0	2	0	1921	1294	876	254	222	188	7.71	5.88	4.70
0	2	200	1747	1282	839	260	215	177	6.88	6.02	4.81
0	4	0	1396	996	769	228	197	169	6.25	5.06	4.56
0	4	200	1587	1055	774	246	210	182	6.43	5.03	4.37
4	0	0	1868	1368	892	269	215	191	6.97	6.36	4.69
4	0	200	1843	1396	880	274	236	178	6.72	5.96	4.93
4	2	0	1793	1341	858	250	237	182	7.34	5.75	4.79
4	2	200	1833	1281	844	250	229	202	7.38	5.70	4.19
4	4	0	1737	1091	777	243	213	183	7.22	5.13	4.29
4	4	200	1624	1188	754	254	209	170	6.41	5.63	4.42
8	0	0	1578	1065	639	256	215	164	6.18	4.96	3.89
8	0	200	1554	1097	649	216	200	190	6.72	5.51	4.48
8	2	0	1512	1011	650	244	213	181	6.22	4.78	3.60
8	2	200	1593	1117	707	224	205	166	7.15	5.51	4.30
8	4	0	1466	943	640	262	197	163	5.62	4.80	3.88
8	4	200	1481	968	671	251	196	167	5.86	4.93	3.99
SEM			112	76.3	65.6	12.9	12.6	11.5	0.50	0.30	0.30
***P*-value**											
Pomegranate peel			0.0007	0.0001	0.0001	0.1636	0.0404	0.1649	0.0335	0.0049	0.0001
Waste oil			0.0046	0.0001	0.0776	0.5382	0.0457	0.2229	0.0332	0.0050	0.5576
α-tocopherol			0.8104	0.3771	0.8819	0.4259	0.8372	0.7664	0.7785	0.2796	0.8426
Pomegranate peel × waste oil			0.4678	0.4197	0.5345	0.0646	0.9393	0.9576	0.6613	0.6654	0.5760
Pomegranate peel × α-tocopherol			0.8873	0.9086	0.6841	0.1038	0.7157	0.8595	0.3671	0.5487	0.7533
waste oil × α-tocopherol			0.8273	0.8480	0.9383	0.3576	0.7684	0.8533	0.7903	0.9264	0.8082
Pomegranate peel × waste oil × α-tocopherol			0.5263	0.8589	0.9924	0.9269	0.7362	0.1692	0.6756	0.7302	0.3815

*SEM* standard error of means, *P*-value probability values.

^a,b^^,c^Means in columns with the same superscript do not differ significantly P < 0.05.

### Intestine morphology

These results (Table 9) indicate that the use of pomegranate peel powder affected villus height, crypt depth, and villus/crypt in broilers at 49 days (p < 0.05). In broilers, the villus height and villus/crypt significantly decreased in the group treated with 8% pomegranate peel powder compared with broilers fed diets with 4% pomegranate peel powder and non-supplemented group in all parts (duodenum, jejunum, and ileum) of the intestine. Also, the use of 8% pomegranate peel powder in diets significantly decreased crypt depth in broilers at 49 days in jejunum parts of the intestine (p < 0.05). A significant effect in villus height and villus/crypt at duodenum and jejunum was observed in the broilers that fed oxidized oil (p < 0.05). Additionally, the results of this study showed that the supplement of 4% oxidized oil in broilers significantly decreased villus height and villus/ crypt at the duodenum and jejunum of the intestine (p < 0.05). In general, no interaction among pomegranate peel powder, waste oil, and α-tocopherol on intestinal morphology has been observed in this study. The current research revealed that the 8% pomegranate peel powder use in diets reduced the villus height at all parts of the intestine, also the difference was significant just between 8% pomegranate peel powder and other groups. In the same manner, using 8% pomegranate peel powder in broiler diets decreased crypt depth in this study. Moreover, feeding 8% of pomegranate peel powder reduced the villi/crypts in the intestine. There is little information about pomegranate peel and intestinal morphology. It seems that these changes are defined by the high amount of tannins that inhibit nutrient absorption and reduce the growth of the body and other tissue in chickens^15,32,43^. It mentioned that the intestine is largely responsible for digestion and absorption of all the principal nutrients, consequently, lower villi height may be related to low digestive capacity^5^. In contrast with our results, researchers indicated that dietary pomegranate peel in quail diets had increased villus height in the jejunum of the intestine^5^.

In this study, 4% waste oil inclusion in diets decreased the broilers' villus height and villi/crypts ratio at 30 and 60% parts of the intestine. This conclusion was comparable with the finding that high peroxide value oil decreased the villus high in birds^6^. The use of high peroxide value oil on intestine morphology was examined by other researchers^43^, who have shown that dietary oxidized oil decreases intestinal mucosa of birds in response to oxidative stress, which can result in low villus height. Similarly, Marchini et al.^48^ observed that oxidative stress in the intestine reduced the villus height on broilers. It has been shown that birds under oxidative stress present smaller crypt depth, mucous area, and villus height of the small intestine, leading to an adverse influence on the intestine function^43^. On the other hand, the inclusion of oxidized oils in broiler diets reduced antioxidants and immune response within the intestinal mucosa. Increasing the amount of ROS in the intestine, results in inflammation and reduces the absorption capacity^43^.

### Meat quality

**Figure 1:**
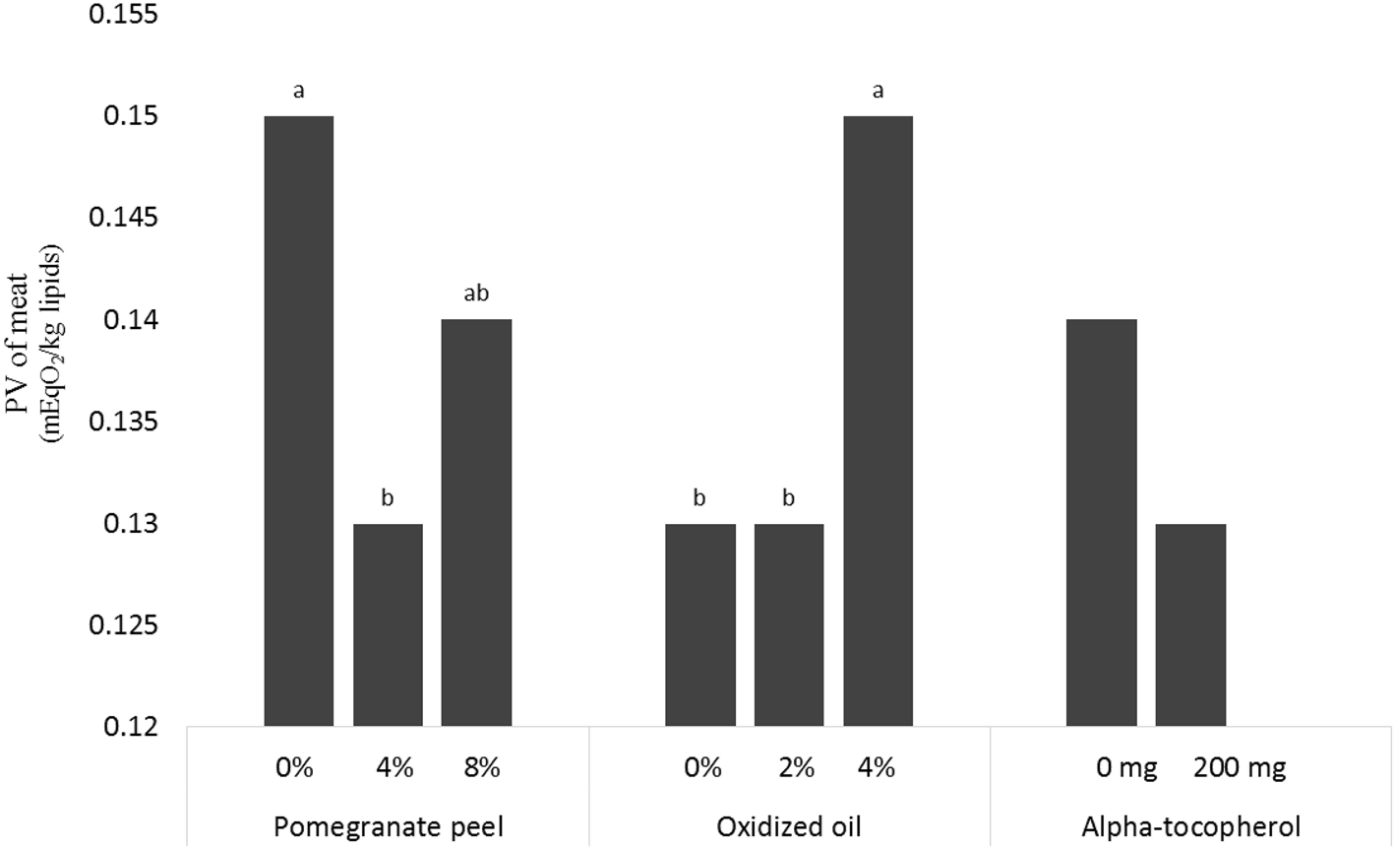
PV concentrations of meat in broiler fed pomegranate peel, oxidized oil and α-tocopherol (main effect). Superscript a, b: means in columns with a different superscript differ significantly p < 0.05. *PV* peroxide value.

The effects of using different levels of pomegranate peel, waste oil, and α-tocopherol in the diet on the peroxide value of meat in broiler chickens are shown in Fig. 1. Results of the experiment indicated that the PV of meat was affected by dietary pomegranate peel and waste oil at the end of the experiment (p < 0.05). The inclusion of 4% pomegranate peel in diets decreased the meat PV of broilers compared to the control groups (p < 0.05). Dietary inclusion of 4% waste oil in diets increased the meat PV when compared to the control group (p < 0.05). No significant interactions have been observed for the meat quality profile. The inclusion of 4% pomegranate peel in diets decreased meat PV and agrees with Ghasemi-Sadabadi et al.^2^ and Abdel Baset et al.^49^. It seems that the inhibitory influence of pomegranate peel on meat oxidation could be due to phenolic compounds, which have an antioxidant influence on lipid oxidation^49^. Similarly, Kishawy et al.^50^ indicated that the use of pomegranate peel extract in diets increased the total phenol and total flavonoid contents of breast muscle in broilers.

It can be concluded that the inclusion of 4% waste oil in diets improved the PV of meat. Researchers also confirmed that dietary waste oil enhanced the PV in the meat of broiler chickens^2,11,51^. Thus, the higher PV of meat has been indicated to be positively correlated with oxidized oil in diets. It had been stated that oxidative damage reduced the quality of poultry meat, including its PV^11^.

## Conclusion

In conclusion, the dietary inclusion of 8% pomegranate peel reduced the growth performance of birds in comparison with the inclusion of 4% pomegranate peel and the control group. The inclusion of 4% pomegranate peel into broiler diets showed no significant effect in comparison with the control group.

The use of 8% pomegranate peel decreased apparent nutrient digestibility in this experiment. Also, the supplementation of 4% waste oil in the diet decreased CF and AME digestibility in broilers. The 4% pomegranate peel and 200 mg/kg α-tocopherol dietary inclusion improved the intestinal population in this experiment. The presence of waste oil in diets reduced the *lactobacilli* population. 4% pomegranate peel in the diet improved the gut morphology of broilers compared with the inclusion of 8% pomegranate peel and the control group. Whereas, the supplementation of 4% waste oil showed lower villus height and crypt depth compared to the other groups. In general, interaction effects among pomegranate peel powder, waste oil, and α-tocopherol have not been observed in this study. The effect of 4% pomegranate peel was decreased on the PV of meat. Also, the inclusion of 4% waste oil into diets reduced meat quality compared with other groups. alpha-tocopherol (200 mg/kg) resulted in improved meat quality.

## Data Availability

The datasets used and/or analyzed during the current study are available from the corresponding author on reasonable request.
